# Single-cell sequencing combined with transcriptome analysis unravels LUM^+^ B cells as key drivers in abdominal aortic aneurysm

**DOI:** 10.3389/fimmu.2026.1836487

**Published:** 2026-07-01

**Authors:** Hanqing Zhang, Junqi Peng, Liman Li, Kexin Han, Yun Yang, Yun Wu, Dengwei Cao, Song Chen

**Affiliations:** 1 Department of Cardiovascular Surgery, Zhongnan Hospital of Wuhan University, Wuhan, China; 2 Hubei Provincial Engineering Research Center of Minimally Invasive Cardiovascular Surgery, Wuhan, China; 3 Wuhan Clinical Research Center for Minimally Invasive Treatment of Structural Heart Disease, Wuhan, China; 4 State Key Laboratory of Metabolism and Regulation inComplex Organisms, Wuhan, China; 5 Department of Laboratory Medicine, West China Hospital of Sichuan University, Chengdu, China; 6 Department of Dermatology, Taikang Tongji (Wuhan) Hospital, Wuhan, China

**Keywords:** abdominal aortic aneurysm, B cells, phenotypic switching, single-cell RNA sequencing, vascular smooth muscle cell

## Abstract

**Background:**

Abdominal aortic aneurysm (AAA) is a life-threatening vascular disease characterized by immune cell infiltration and vascular remodeling. B cells have been implicated in AAA pathogenesis, yet their specific roles and molecular mediators remain incompletely understood. This study aimed to investigate the immune microenvironment of AAA and elucidate the functional role of LUM in B cells using integrated multi-omics and experimental approaches.

**Methods:**

We integrated single-cell RNA sequencing (scRNA-seq) and bulk transcriptome data from GEO datasets (GSE183464, GSE226492). Key computational analyses included cell clustering, trajectory inference (CytoTRACE2, Monocle2), cell communication (CellChat), and machine learning-based feature selection (LASSO and SVM). Experimentally, primary human B cells were isolated and subjected to lentivirus-mediated LUM knockdown or overexpression, followed by Transwell co-culture with primary human aortic vascular smooth muscle cells (VSMCs). LUM expression in B cells and plasma was further validated in clinical samples, and its correlation with aneurysm diameter was analyzed.

**Results:**

Single-cell analysis identified B cells as a significantly altered immune population in AAA with enhanced communication to VSMCs. CD79A and LUM were identified as key signature genes in B cells. LUM was upregulated at both mRNA and protein levels in AAA tissues and specifically enriched in B cells. Functional experiments demonstrated that LUM expression in B cells promoted VSMC phenotypic switching toward a synthetic phenotype (upregulated OPN and downregulated contractile markers). Knockdown of LUM in B cells attenuated this effect, whereas overexpression enhanced it. Clinically, LUM protein levels in B cells and plasma increased with larger aneurysm diameter and positively correlated with maximum AAA diameter.

**Conclusions:**

This study reveals that LUM^+^ B cells play a critical role in AAA by promoting VSMC phenotypic switching. The combination of single-cell transcriptomics, functional validation, and clinical correlation establishes LUM in B cells as both a mechanistic contributor and a potential biomarker for AAA severity. These findings provide new insights into B cell-mediated vascular remodeling and highlight LUM as a promising therapeutic target.

## 1 Introduction

Abdominal aortic aneurysm (AAA) is a life-threatening vascular condition characterized by the localized and irreversible dilation of the abdominal aorta. The pathogenesis of AAA is attributed to multiple risk factors, such as hypertension, atherosclerosis, smoking, advanced age, male gender, and genetic predisposition (1–4). The most catastrophic outcome of AAA progression is rupture, which carries an exceedingly high mortality rate of 67%–94% (2, 5). Currently, the primary treatment strategies for larger, high-risk aneurysms are open surgical repair or endovascular aortic repair. However, these invasive procedures are associated with significant operative risks and potential complications, including endoleak, infection, and thrombosis (3, 6). This critical limitation underscores the urgent need for the development of novel, non-surgical therapeutic alternatives.

Immune dysregulation has been recognized as a key pathological mechanism across a wide range of diseases, including inflammatory bowel disease, renal cell carcinoma, and multiple cancer types, where aberrant immune cell infiltration and disordered immune signaling drives disease progression (7–9). Growing evidence indicates that this pathological role of immune dysregulation extends beyond classical immune-mediated diseases to vascular pathologies, and AAA is a well-characterized example in which immune cell infiltration and inflammatory signaling are recognized as central contributors to disease initiation and progression (1, 2, 10, 11).

Decades of research have established that the pathogenesis of AAA involves a complex interplay of inflammatory gene networks and immune dysregulation, in which the coordinated dysregulation of multiple inflammatory pathway drives disease progression and represents a potential source of therapeutic targets (2, 12). Key pathological features include inflammatory cell infiltration, extracellular matrix (ECM) degradation, and vascular smooth muscle cell dysfunction (2, 13), all of which are closely associated with inflammatory and immune cell infiltration in both the adventitia and intima (14). The formation of AAA involves the coordinated action of both innate and adaptive immune cells. It begins with the initial accumulation of neutrophils within the vascular wall, marking the onset of aortic wall destruction (11). This is followed by the infiltration of early myeloid cells, recognized as a hallmark of AAA progression (1, 10, 15). Subsequently, the recruitment of macrophages and monocytes, accompanied by the release of related inflammatory factors, together with the activation of adaptive immunity (10, 15, 16), collectively contribute to vascular remodeling and weakening of the aortic wall, ultimately leading to AAA formation.

Although extensive immune cell infiltration has been recognized as a core pathological feature of AAA, effective immune-targeted therapies remain unavailable. A critical barrier to therapeutic development lies in the incomplete understanding of adaptive immune regulation in AAA, particularly the mechanisms by which specific immune cell population contribute to disease pathogenesis. Among adaptive immune components, B cells have received comparatively less attention despite accumulating evidence of their pathological relevance. Several studies have identified substantial B-cell infiltration within the adventitia of human AAA (17–19), indicating their significant involvement in disease pathogenesis (20). Experimental evidence further confirms the functional importance of B cells: in B-cell-deficient muMT murine models, Matrix Metalloproteinase-9 (MMP-9) expression was significantly reduced and AAA severity was attenuated (21, 22). Furthermore, inhibition of Syk, a key signaling molecule in the B-cell receptor pathway, effectively suppressed aneurysm growth, alleviated vascular inflammation, and decreased immunoglobulin deposition within AAA tissues (23, 24). Another study reported that in AAA tissue, B cells were found in close spatial proximity to macrophages and potentially exacerbated pathological progression by impairing macrophage efferocytosis (25). These findings collectively demonstrate that B cells not only directly promote AAA development through their own effector activities, but also indirectly modulate other immune populations via complex intercellular communication, thereby playing a multifaceted role in AAA pathology.

In this study, we employed an integrated approach combining single-cell RNA sequencing (scRNA-seq) and bulk transcriptome sequencing to profile immune cells in AAA tissues. Our objectives were to: (1) delineate the landscape of immune cell clusters and characterize the intercellular communication networks within the AAA microenvironment; (2) investigate the specific role of B cells by identifying B cell-related genes and their associated signaling pathways; and (3) validate the pathological relevance of key findings through histological analysis. This comprehensive strategy aims to identify novel indicators for the early diagnosis of AAA and to evaluate the therapeutic potential of B cells, thereby laying the groundwork for future development of B cell-targeted interventions.

## 2 Materials and methods

### 2.1 Enrollment of study participants and collection of tissue samples

The study protocol for human tissue sample collection was approved by the Ethics Committee at Zhongnan Hospital of Wuhan University (No. 2026011K). Prior to enrollment, all participants or legally authorized representatives of organ donors provided written informed consent. All experiments involving human tissue samples were conducted in strict compliance with applicable guidelines and regulations. Control aortic samples were procured from individuals who underwent heart transplants, while diseased aortic tissue samples were obtained from patients diagnosed with AAA. Patients with abdominal aortic dissection were excluded from the study.

### 2.2 Data acquisition

The GEO database (https://www.ncbi.nlm.nih.gov/geo/info/datasets.html), also known as GENE EXPRESSION OMNIBUS, is a gene expression database created and maintained by the National Center for Biotechnology Information (NCBI). We downloaded mRNA expression data related to abdominal aortic aneurysm (GSE183464) from the NCBI GEO public database. A total of 14 samples were collected, including 7 diseased and 7 control samples. We also downloaded single-cell data files for GSE226492, including sample data with complete single-cell expression profiles for single-cell analysis. Three diseased and three control samples were used.

### 2.3 Single-cell data quality control

First, expression profiles were read in using the Seurat package (version 4.3.0). Cells were filtered based on the total number of UMIs per cell, the number of expressed genes, and the mitochondrial expression percentage per cell. The mitochondrial gene expression percentage refers to the percentage of mitochondrial gene expression relative to the total expression of all genes. Cells with a high mitochondrial gene expression percentage have low RNA expression, indicating that these cells are undergoing apoptosis. We performed quality control using the median absolute deviation (MAD). Generally, if a variable is more than 3 MADs away from the median, it is considered an outlier and needs to be removed, completing cell quality control.

### 2.4 Single-cell data dimensionality reduction, clustering, and cell annotation

We performed global normalization using the LogNormalize method, which scales the total expression of each cell to 10,000 using a scaling factor s0, followed by log transformation. Cell cycle scores were calculated with the CellCycleScoring function, and highly variable genes were identified using FindVariableFeatures (object = scRNA, selection.method = “vst”, nfeatures = 2000). To mitigate unwanted sources of variation, including the proportions of mitochondrial and ribosomal genes and cell cycle effects, we applied the ScaleData (object = scRNA, vars.to.regress = c (“percent.mt”, “percent.ribo”, “S.Score”, “G2M.Score”)). Linear dimensionality reduction was carried out via RunPCA on the expression matrix, and significant principal components were selected for downstream analyses. Batch effects were corrected using Harmony, and nonlinear dimensionality reduction was performed with RunUMAP (Uniform Manifold Approximation and Projection). For cell type annotation, we combined manual curation based on the CellMarker and PanglaoDB databases and literature review with automated annotation using the SingleR package, thereby identifying cell types and their corresponding marker genes across tissues.

### 2.5 Isolation and culture of primary human aortic vascular smooth muscle cells

Primary human aortic vascular smooth muscle cells (VSMCs) were isolated from aortic tissue specimens using the tissue explant adherence method. Aortic samples were washed thoroughly with ice-cold PBS containing 1% penicillin-streptomycin. The adventitia and intima layers were carefully removed, and the remaining media was cut into small pieces (approximately 1–2 mm³). These tissue explants were placed in 6-well plates pre-coated with 0.1% gelatin and cultured in smooth muscle cell-specific growth medium (SmGM™-2 BulletKit, Lonza) supplemented with 5% fetal bovine serum (FBS), recombinant human epidermal growth factor (rhEGF), recombinant human basic fibroblast growth factor (rhFGF-B), insulin, and antibiotics. VSMCs migrated out from the explants within 7–10 days. Cells were passaged upon reaching 80–90% confluence, and VSMCs between passages 3 and 6 were used for experiments.

### 2.6 Isolation of peripheral blood B cells, lentiviral manipulation, and functional assays

Peripheral blood mononuclear cells (PBMCs) were isolated from AAA patients and healthy controls by Ficoll-Paque density gradient centrifugation. CD79^+^ B cells were purified by fluorescence-activated cell sorting (FACS) using APC-conjugated anti-human CD79A antibody. The purity of sorted B cells was consistently greater than 95%. Primary human peripheral blood B cells (HPBC) were cultured in RPMI 1640 medium supplemented with 10% FBS, 1% penicillin-streptomycin, and 50 ng/mL recombinant human IL-4.

For lentiviral manipulation, HPBC were transduced with lentivirus carrying shRNA targeting human LUM (sh-LUM) or a LUM overexpression construct (LUM^OE^) at a multiplicity of infection (MOI) of 20 in the presence of 8 μg/mL polybrene. Transduction efficiency and modulation of LUM expression were verified by qRT-PCR and Western blot 72 hours after transduction.

For Western blot analysis of LUM in B cells, freshly sorted CD79^+^ B cells from AAA patients and healthy controls were lysed in RIPA buffer containing protease and phosphatase inhibitors. Protein concentration was determined using the Bradford assay. Equal amounts of protein were separated by SDS-PAGE, transferred to PVDF membranes, and probed with anti-LUM and anti-GAPDH antibodies. Bands were visualized using enhanced chemiluminescence and quantified using ImageJ software.

For plasma LUM measurement, peripheral blood was collected into EDTA tubes and centrifuged at 1,500 × g for 15 minutes at 4 °C. Plasma was carefully collected and stored at −80 °C until use. LUM protein concentration in plasma was determined using a commercial human Lumican ELISA kit (MEI MIAN) according to the manufacturer’s instructions.

For co-culture experiments, VSMCs were seeded in the lower chamber of 6-well Transwell plates (0.4 μm pore size) at a density of 1 × 10^5^ cells per well and allowed to adhere overnight. Lentivirus-transduced B cells were added to the upper chamber at the same density. After 48 hours of co-culture, VSMCs in the lower chamber were harvested for Western blot analysis of phenotypic markers (OPN, αSMA, CNN, and SM22α). In parallel, the culture supernatant from the upper chamber was collected, and the concentration of LUM protein was measured using ELISA as described above.

### 2.7 MiloR analysis

MiloR (version 2.5.1) is a graph-based differential abundance analysis algorithm that constructs a cell neighborhood graph, partitions cells into local neighborhoods, and counts the number of cells within each sample that fall within these neighborhoods. MiloR then uses a generalized linear model to compare cell abundance across different conditions within each neighborhood, identifying regions where cell composition changes significantly under specific biological conditions, thereby revealing differential cell abundance within subpopulations or continuous states.

### 2.8 Cell potency and trajectory analysis

To delineate the developmental potential and differentiation trajectories within cell subpopulations, we performed integrated analyses using CytoTRACE2 (version 0.3.1) and Monocle2 (version 2.30.0). Cellular developmental potency was assessed with CytoTRACE2, which assigned a continuous potency score (CytoTRACE2_Score) to each cell. These scores were visualized on UAP plots and incorporated into subsequent trajectory analysis. Pseudotemporal ordering was conducted using the Monocle2 package. This method reconstructed the differentiation trajectory by calculating cell-to-cell similarities and ordering cells along a minimum spanning tree based on transcriptional progression. Genes exhibiting the most significant variation along the inferred pseudotime were identified for further analysis.

### 2.9 Cell-cell communication analysis

Cell-cell communication was quantitatively inferred and analyzed using CellChat (version 2.1.2), which models signaling networks based on the expression of ligand-receptor pairs. The normalized single-cell expression matrix and the annotated cell subtype labels were provided as input. The tool was applied to both control and disease groups to predict major signaling inputs and outputs for each cell type, and to calculate interaction probabilities (weights) and counts. The inferred communication networks were compared between the two groups to identify altered signaling pathways. The results were visualized to depict the number and strength of interactions between cell populations.

### 2.10 Lasso regression and random forest feature selection

Single-cell RNA sequencing data are inherently noisy and sparse. To ensure the reliability and biological relevance of the identified differentially expressed genes (DEGs), we screened differentially expressed genes with | log2FC |>2&puvaldj<0.05 in key cells as candidate genes. This threshold isolates cell-type-specific core marker genes and prioritizes key regulators with distinct biological functions. Specifically, |log2FC| > 2 ensures that gene expression has a sufficiently large biological change magnitude (≥4-fold) which provides a robust foundation for downstream experimental validation and mechanistic studies. We then used Lasso logistic regression and the Support Vector Machine (SVM) algorithm to select features for disease diagnostic biomarkers. The Lasso algorithm was implemented using the “glmnet” package (version 4.1-8). Furthermore, Support Vector Machine-Recursive Feature Elimination (SVM-RFE), a machine learning method based on SVM, identified the optimal variables by removing SVM-generated feature vectors. A SVM model was then constructed using the “e1071” package (version 1.7-16) to further identify the diagnostic value of these biomarkers. RandomForest (version 4.7-1.1) was used to build classification models and evaluate key features.

### 2.11 Immune microenvironment analysis

To quantify the immune cell composition within the tissue microenvironment, we estimated the relative abundances of 22 human immune cell types using the CIBERSORT algorithm (version 0.1.0). This analysis was performed on bulk RNA-seq dataset from the abdominal aortic aneurysm cohort (GSE183464). CIBERSORT employs a support vector regression model to deconvolute the gene expression matrix, utilizing a predefined signature matrix of 547 genes that distinguishes various immune cell phenotypes, including T cells, B cells, plasma cells, and myeloid subsets.

### 2.12 GSEA analysis

Based on the expression of key genes, samples were divided into high- and low-expression groups. GSEA was used to further analyze signaling pathway differences between the two groups. A background gene set, version 7.0, was downloaded from the MsigDB database as an annotation gene set for subtype pathways. Differential expression of pathways between groups was analyzed, and significantly enriched gene sets (adjusted p-value less than 0.05) were ranked based on consistency scores. GSEA analysis is often used to closely integrate disease classification with biological significance.

### 2.13 GSVA analysis

Gene Set Variation Analysis (GSVA) was performed to assess the enrichment of specific biological pathways in an unsupervised, non-parametric manner. This method transforms the gene-level expression matrix into a pathway-level enrichment score matrix, allowing for the characterization of sample-specific biological functions. Gene sets of interest were obtained from the Molecular Signatures Database (MSigDB). The GSVA algorithm was then applied to our transcriptome data to compute a single enrichment score for each gene set and each sample, thereby quantifying the activity of diverse biological processes.

### 2.14 Molecular docking

Based on key genes, corresponding protein 3D structures were obtained from the Alphafold database (https://alphafold.com/). Key gene drug predictions were performed using the CTD database (https://www.ctdbase.org/) to obtain relevant key compounds. Drug compound structures were then obtained from the PubChem database (https://pubchem.ncbi.nlm.nih.gov/). Molecular docking was performed using AutoDock software (version 1.5.7), with nine docking runs. The docking result with the lowest binding energy, excluding the first reference conformation, was selected for display. The results were then imported into PYMOL for visualization, showing the binding sites of the small molecule to the protein.

### 2.15 Molecular dynamics simulations

Molecular dynamics (MD) simulations were performed using Gromacs 2023, with the GAFF force field for small molecules and the AMBER14SB force field and TIP3P water model for proteins. The protein and small molecule ligand files were merged to construct the complex simulation system. MD simulations were conducted at constant temperature and pressure with periodic boundary conditions. All hydrogen bonds involved were constrained using the LINCS algorithm, with an integration step of 2 fs. Electrostatic interactions were calculated using the (Particle-mesh Ewald) PME method with a cutoff of 1.2 nm. Non-bonded interactions were calculated with a cutoff of 10 Å, updated every 10 steps. The simulation temperature was maintained at 298 K using the V-rescale temperature coupling method, and the pressure was maintained at 1 bar using the Berendsen method. At 298 K, equilibrium simulations of NVT and NPT were performed for 100 ps, and MD simulations of the complex were performed for 100 ns, with conformations saved every 10 ps. After completion, the simulation trajectories were analyzed using VMD and pymol. Stability was assessed using RMSD, Rg, RMSF, and Buried SASA analysis.

### 2.16 Quantitative real-time polymerase chain reaction

Total RNA extraction from AAA and healthy aorta tissues was performed using the HiPure Total RNA Mini Kit (Cat. #R4111-03, Magen, China). Subsequently, reverse transcription was carried out using the ReverTra Ace qPCR RT Kit (Toyobo, China). QRT-PCR was conducted using iQTM SYBR^®^ Green Supermix from Bio-Rad in the USA. **Supplementary Table 1** lists the primer sequences used. The fold enrichment was determined using the 2−ΔΔCt method and normalized to GAPDH expression.

### 2.17 Western blot

AAA and normal aortic tissues lysates and protein samples were prepared with RIPA buffer, protease inhibitor, and phosphatase inhibitor (Sigma-Aldrich, USA). The Bradford protein assay (Bio-Rad, Germany) was used to evaluate the protein concentration. Western blot analysis was conducted following the fractionation of total protein samples through 7.5-15% SDS-PAGE. Immunoreactive bands were visualized using an enhanced chemiluminescence kit (Bio-Rad, USA) and were then detected using a Molecular Imager ChemiDoc XRS + Imaging System (Bio-Rad, USA). **Supplementary Tables 2, 3** list the primary antibodies and secondary antibodies used, respectively.

### 2.18 Immunohistochemical staining

Surgical tissue specimens were fixed with formalin to for paraffin-embedded. IHC analyses were performed on 4 μm thick sections. Briefly, each slide was incubated with primary antibody against LUM overnight after a series of procedures (de-paraffin, antigen retrieval, rinse). This was followed by incubation with the anti-rabbit IgG-HRP antibody for 30 min. The membrane was then washed five times with TBST and enriched with the brown color of DAB Enhancer (Dako, China). The LUM expression was evaluated by three experienced pathologists. We used phase contrast microscope to analyze IHC sections.

### 2.19 Statistical analysis

Statistical analyses were conducted using R software (version 4.3.0). Continuous variables between two groups were compared using the Wilcoxon rank-sum test or Kruskal-wallis test. Correlations were evaluated by calculating Spearman’s correlation coefficients. A two-sided p-value of less than 0.05 was defined as statistically significant.

## 3 Results

### 3.1 Single-cell data quality control, dimensionality reduction, clustering, and cell annotation

Considering the data quality of multiple samples, the captured cells with abnormal values will be filtered. Finally, a total of 63,335 cells were retained, and the violin plots and scatter plots after quality control were shown in **Supplementary Figures 1A, B**. Following the identification of 2,000 highly variable genes, the data were scaled and centered. Subsequently, principal component analysis (PCA) was performed, followed by batch effect correction using Harmony. Finally, the cells were visualized in two dimensions with Uniform Manifold Approximation and Projection (UMAP) (**Supplementary Figures 1C, D**).

**Figure 1:**
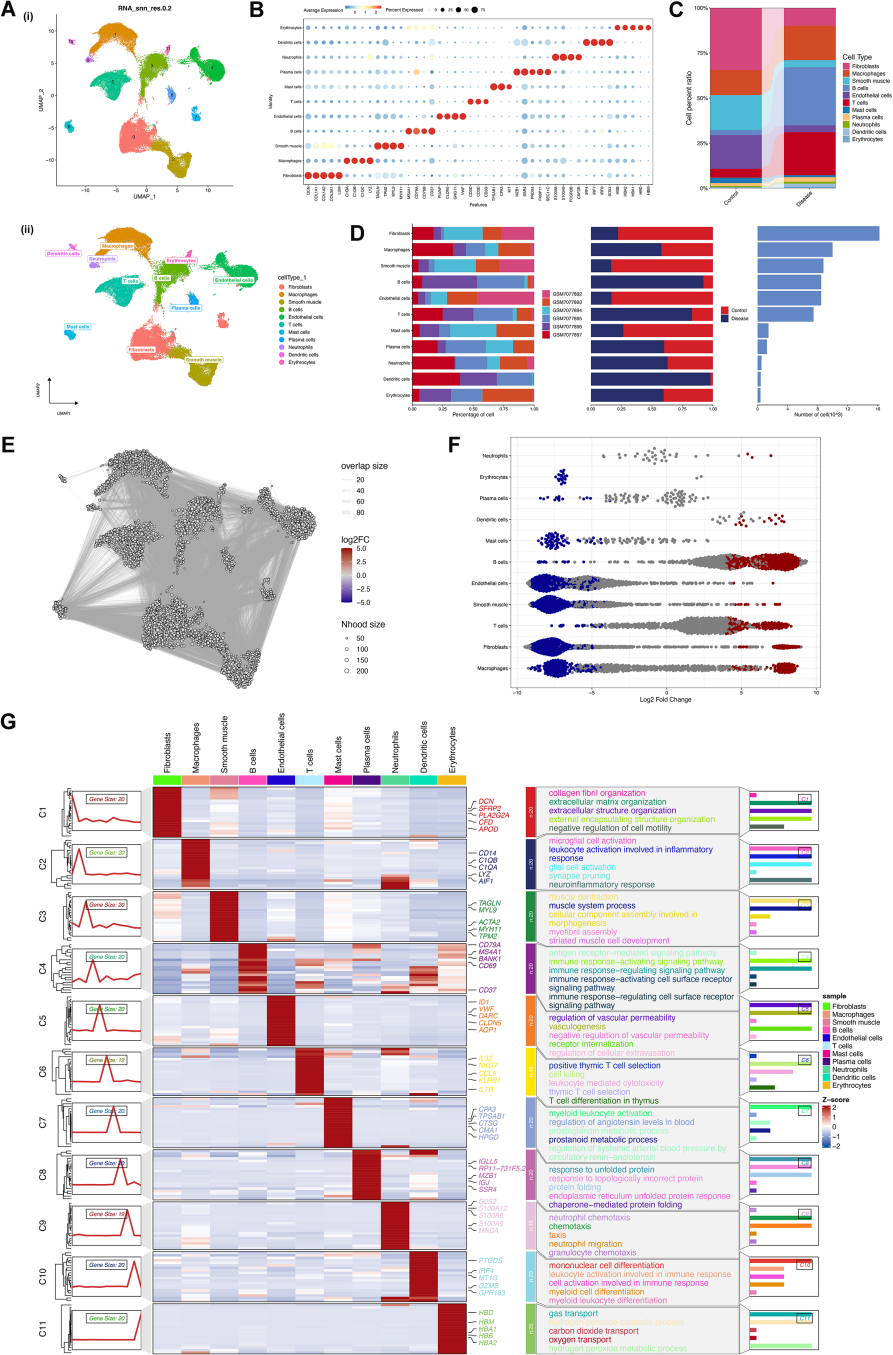
Single-cell clustering, cell annotation and spatial mapping of key cell subpopulations. **(A)** Single-cell clustering and cell annotation. **(B)** The bubble plot visualizes the top highly expressed genes for each cell type. **(C, D)** The stacked bar plot reveals pronounced differences in cellular composition across samples. **(E)** A neighborhood network graph visualizes the results of differential abundance testing. **(F)** A beeswarm plot displays the distribution of differential abundance across neighborhoods. **(G)** Subgroup difference and enrichment analysis.

After dimensionality reduction using UMAP, a total of 12 subgroups were obtained. Then, we annotated cell types by cross-referencing the top 100 marker genes (by fold change) per cluster with known cellular markers, assigning the top five matches as the cluster identity. These clusters were annotated as 11 principal cell populations: fibroblasts (cluster 7 and 0), macrophages (cluster 1), smooth muscle cells (cluster 2), B cells (cluster 3), endothelial cells (cluster 4), T cells (cluster 5), mast cells (cluster 6), plasma cells (cluster 8), neutrophils (cluster 9), dendritic cells (cluster 10), and erythrocytes (cluster 11) (**Figure 1A**). The bubble plot visualizes the top highly expressed genes for each cell type, demonstrating their specific enrichment in corresponding populations (**Figure 1B**). Complementing this, the stacked bar plot reveals pronounced differences in cellular composition across samples. Specifically, cell types such as fibroblasts, endothelial cells, smooth muscle cells, and mast cells were significantly enriched in normal abdominal aorta. In contrast, macrophages, B cells, plasma cells, neutrophils, and dendritic cells were predominantly found in abdominal aortic aneurysm (AAA) samples (**Figures 1C, D**). Notably, while fibroblasts were the most abundant population, B cells were markedly enriched in AAA, motivating our subsequent focus on their subpopulation dynamics. (**Figure 1D**).

### 3.2 Spatial mapping of B cells subpopulation

The spatial abundance changes of cellular neighborhoods between the healthy control group (HE) and the wild-type group (WT) groups across two time points were assessed using MiloR, enabling identification of cell states exhibiting significant differences in spatial or compositional dimensions. A neighborhood network graph visualizes the results of differential abundance testing, where each node represents a cell neighborhood constructed from a KNN graph, with node size proportional to the number of cells contained (**Figure 1E**). Complementarily, a beeswarm plot displays the distribution of differential abundance (logFC) across neighborhoods, colored by statistical significance. This analysis revealed that B cell neighborhoods underwent marked abundance changes between experimental conditions, identifying them as a key cell subpopulation in the observed response (**Figure 1F**).

Subsequently, subgroup enrichment analysis was performed, and ClusterGVis was used to visualized the average expression profiles of each subgroup (**Figure 1G**). The results showed that the differential genes of the key cell subgroup B cells were enriched in pathways such as antigen receptor-mediated signaling pathway, immune response-activating signaling pathway, immune response-regulating signaling pathway, and immune response-activating cell surface receptor signaling pathway. These results suggest that B cell-mediated immune responses play a critical role in AAA pathogenesis.

### 3.3 CytoTRACE defines the starting point of cell development and pseudo-time series analysis

**Figure 2:**
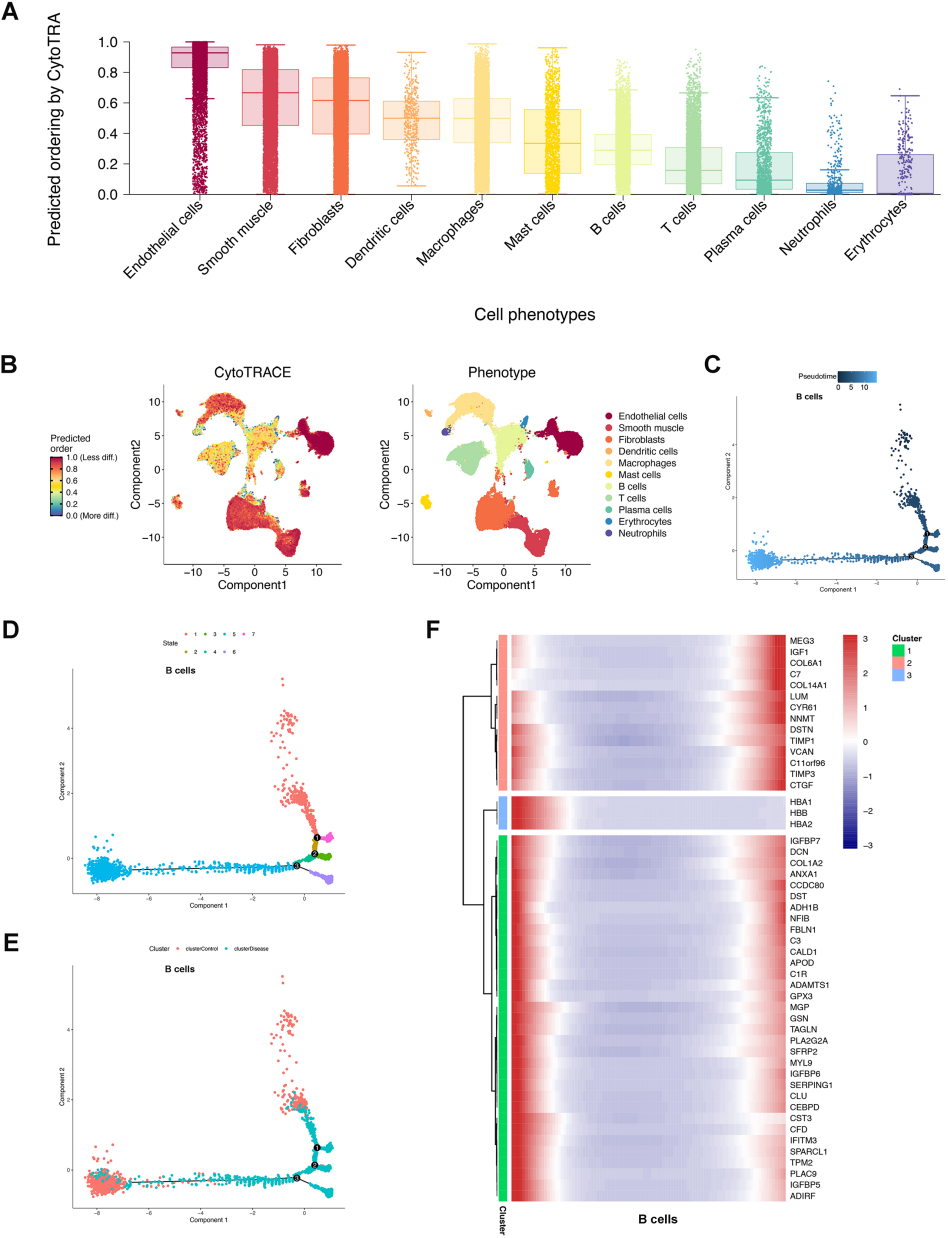
B Cell development and pseudo-time series analysis. **(A, B)** The CytoTRACE2 analysis. **(C–E)** Pseudotemporal trajectory reconstruction using Monocle2. **(F)** Analysis of genes dynamically expressed along the pseudotime axis.

The CytoTRACE2 analysis revealed a pronounced reduction in developmental potential among B cells within the disease group, indicating a more differentiated state (**Figures 2A, B**). This was further supported by pseudotemporal trajectory reconstruction using Monocle2, which positioned B cells predominantly in the middle to late phases of the inferred differentiation path. Notably, B cells from the disease group were enriched in trajectory states associated with advanced differentiation, whereas control B cells were more abundant in earlier states (**Figures 2C–E**). Analysis of genes dynamically expressed along the pseudotime axis identified three distinct expression clusters (**Figure 2F**). Among these, B-cell–related genes were significantly enriched in the late-phase cluster, which also included MEG3, IGF1, and COL6A1. This pattern suggests that B cells undergo transcriptional maturation during disease progression and may actively contribute to extracellular matrix remodeling and inflammatory signaling in the late stages of AAA.

### 3.4 Cell communication

**Figure 3:**
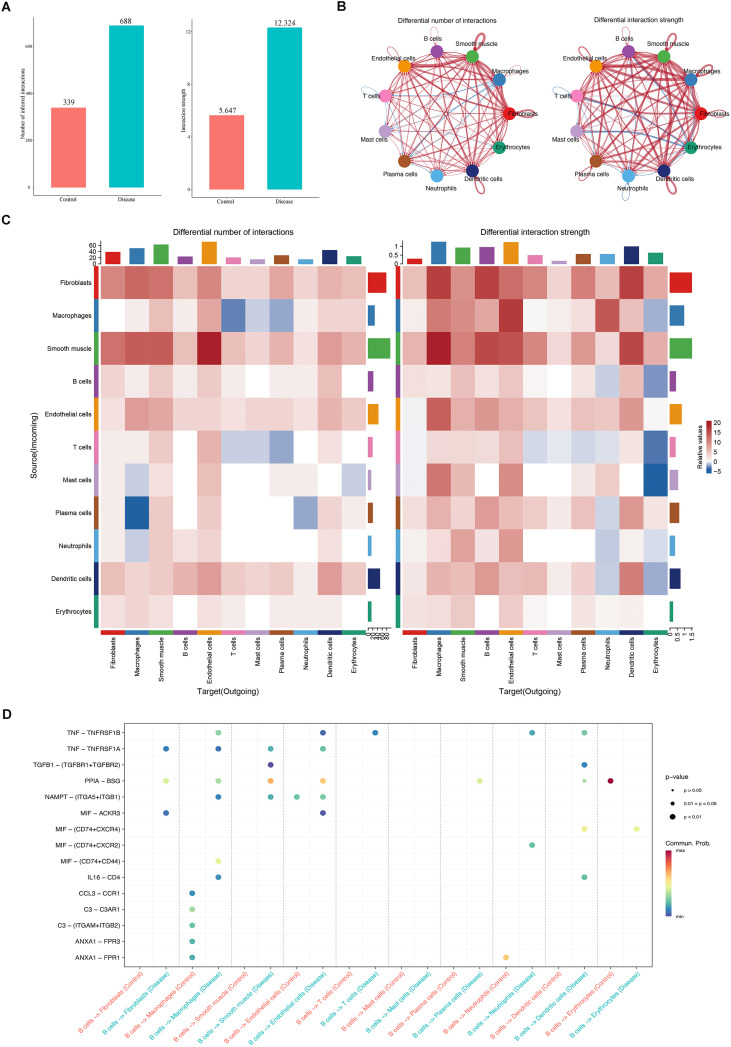
Cell communication. **(A)** AAA tissues exhibit a greater number of intercellular communication links compared to controls. **(B)** The circos plot reveals that B cells receive signals from almost all major cell types present in the abdominal aortic wall. **(C)** Communication between B cells and fibroblasts, endothelial cells, dendritic cells, and smooth muscle cells appears particularly frequent. **(D)** Analysis identified several significantly upregulated ligand-receptor pairs in AAA (B cells-fibroblasts, B cells-smooth muscle cells, B cells-endothelial cells).

B cells, as key immune cells, primarily influence disease progression by secreting immune mediators such as inflammatory factors and antibodies, which in turn modulate other cell types. To better understand their role, we further investigated the communication networks between B cells and other cells in the context of AAA. As depicted in **Figure 3A**, AAA tissues exhibit a greater number of intercellular communication links compared to controls, underscoring the importance of cellular crosstalk in disease pathogenesis. The circos plot (**Figure 3B**) reveals that B cells receive signals from almost all major cell types present in the abdominal aortic wall. Notably, communication between B cells and fibroblasts, endothelial cells, dendritic cells, and smooth muscle cells appears particularly frequent (**Figure 3C**). Given the well-established roles of these cell types in AAA, our findings suggest that B cells may act as critical intermediaries in their pathological functions during disease progression.

Further analysis identified several significantly upregulated ligand–receptor pairs in AAA. Between B cells and fibroblasts, the signals mediated by TNF–TNFRSF1A, PPIA–BSG, and MIF–ACKR3 were markedly enhanced. In B cell–smooth muscle cell interactions, TNF–TNFRSF1A, TGFB1, PPIA–BSG, and NAMPT–(ITGA5+ITGB1) were notably elevated. Similarly, between B cells and endothelial cells, TNF–TNFRSF1B, TNF–TNFRSF1A, PPIA–BSG, and MIF–ACKR3 were significantly upregulated during disease progression. Among these, TNF–TNFRSF1A and PPIA–BSG were consistently upregulated across all three interaction pairs, highlighting their potential as key signaling axes through which B cells exert their influence in AAA (**Figure 3D**). These findings highlight B cells as central communicators in the AAA immune microenvironment, with TNF–TNFRSF1A and PPIA–BSG emerging as the most consistently activated signaling axes.

### 3.5 CD79A and LUM are co-expressed in B cells and associated with AAA progression

**Figure 4:**
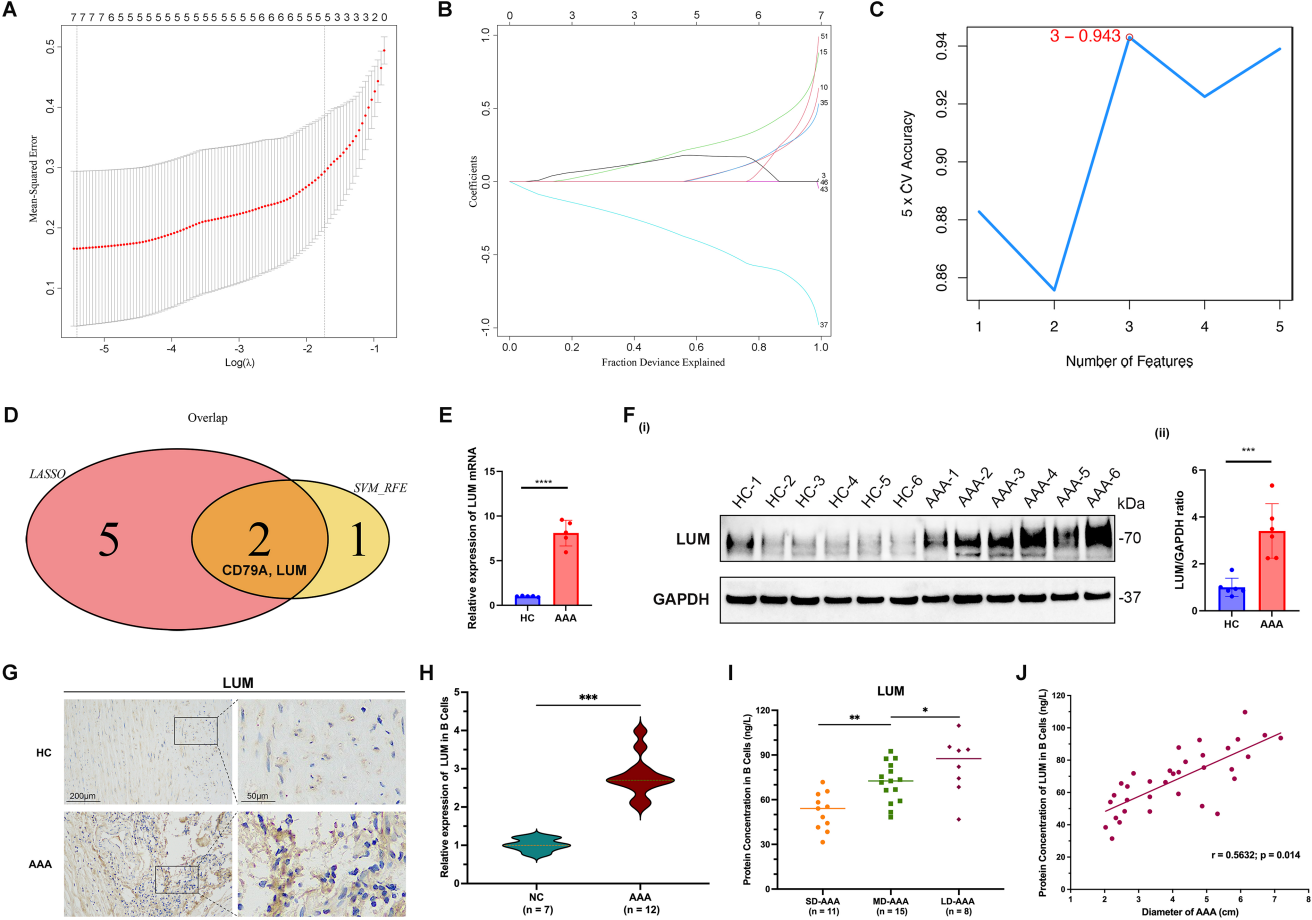
Identification of AAA signature genes in B cells. **(A, B)** LASSO regression identified 7 signature genes in B cells. **(C)** The SVM model selected 3 signature genes in B cells. **(D)** The intersection of these two gene lists yielded two key genes: CD79A and LUM. **(E)** qRT-PCR analysis reaffirms the elevated transcriptome level of LUM in human AAA compared to healthy control. **(F, G)** Western blot and IHC analyses corroborates the abundant translational level of LUM in human AAA compared to healthy control. **(H)** ELISA measurement of LUM protein concentration in plasma from normal controls (NC) and AAA patients(n = 7 for NC, n = 12 for AAA). **(I)** ELISA measurement of LUM protein concentration in B cells from AAA patients stratified by aneurysm diameter into small-diameter (SD-AAA), medium-diameter (MD-AAA), and large-diameter (LD-AAA) groups. **(J)** Correlation analysis between LUM protein concentration in B cells and maximum AAA diameter. A significant positive correlation was observed. ^*^p<0.05, ^**^p<0.01, ^***^p<0.001, ^****^p<0.0001.

To identify key genes implicated in AAA, we first screened for differentially expressed genes in B cells using thresholds of |log2FC| > 2 and an adjusted p-value < 0.05. This candidate gene set was subsequently subjected to feature selection using both Least Absolute and Selection Operator (LASSO) regression and Support Vector Machine (SVM) algorithms. LASSO regression identified 7 signature genes (**Figures 4A, B**), while the SVM model selected 3 features (**Figure 4C**). The intersection of these two gene lists yielded a final set of two key genes: CD79A and LUM (**Figure 4D**), which were prioritized for all subsequent analyses.

Based on bulk-seq deconvolution, AAA samples had higher levels of naïve B cells and neutrophils, but fewer monocytes compared to controls. Correlation analysis showed that CD79A was associated with multiple immune cell types, including naïve B cells, M0 macrophages, and activated CD4 memory T cells. In contrast, LUM showed a strong positive correlation specifically with neutrophil infiltration. Together, these findings suggest that CD79A and LUM may play distinct yet complementary roles in the AAA immune microenvironment.

To validate these findings, we examined LUM expression at both the transcriptional and protein levels in clinical samples. qRT-PCR confirmed significant upregulation of LUM mRNA in AAA tissues compared to healthy controls (**Figure 4E**). This was further supported at the protein level by Western blot analysis, which showed markedly increased LUM protein abundance in AAA samples (**Figure 4F**). Immunohistochemical staining additionally demonstrated elevated LUM expression in AAA tissues, with positive staining notably enriched in infiltrating immune cells (**Figure 4G**).

To further investigate LUM expression specifically within B cells and its clinical relevance, we isolated CD79A^+^ B cells from peripheral blood of AAA patients and healthy controls. LUM mRNA expression in B cells was significantly higher in AAA patients compared with normal controls (**Figure 4H**). Moreover, when AAA patients were stratified by maximum aneurysm diameter into small-diameter (SD-AAA), medium-diameter (MD-AAA), and large-diameter (LD-AAA) groups, LUM protein concentration in B cells (measured by ELISA) showed a stepwise increase with larger aneurysm size (**Figure 4I**). Importantly, LUM protein levels in B cells positively correlated with maximum AAA diameter (**Figure 4J**). These results indicate that LUM is not only upregulated in AAA but is also specifically enriched in B cells and associated with disease severity.

### 3.6 LUM expression in B cells promotes vascular smooth muscle cell (VSMC) phenotypic switching to a synthetic phenotype

Vascular smooth muscle cell (VSMC) phenotypic switching from a contractile to a synthetic phenotype plays a critical role in the initiation and progression of AAA. To investigate whether LUM expressed in B cells contributes to this pathological process, we performed functional experiments using lentivirus-mediated shRNA knockdown and overexpression of LUM in primary human peripheral blood B cells (HPBC).

**Figure 5:**
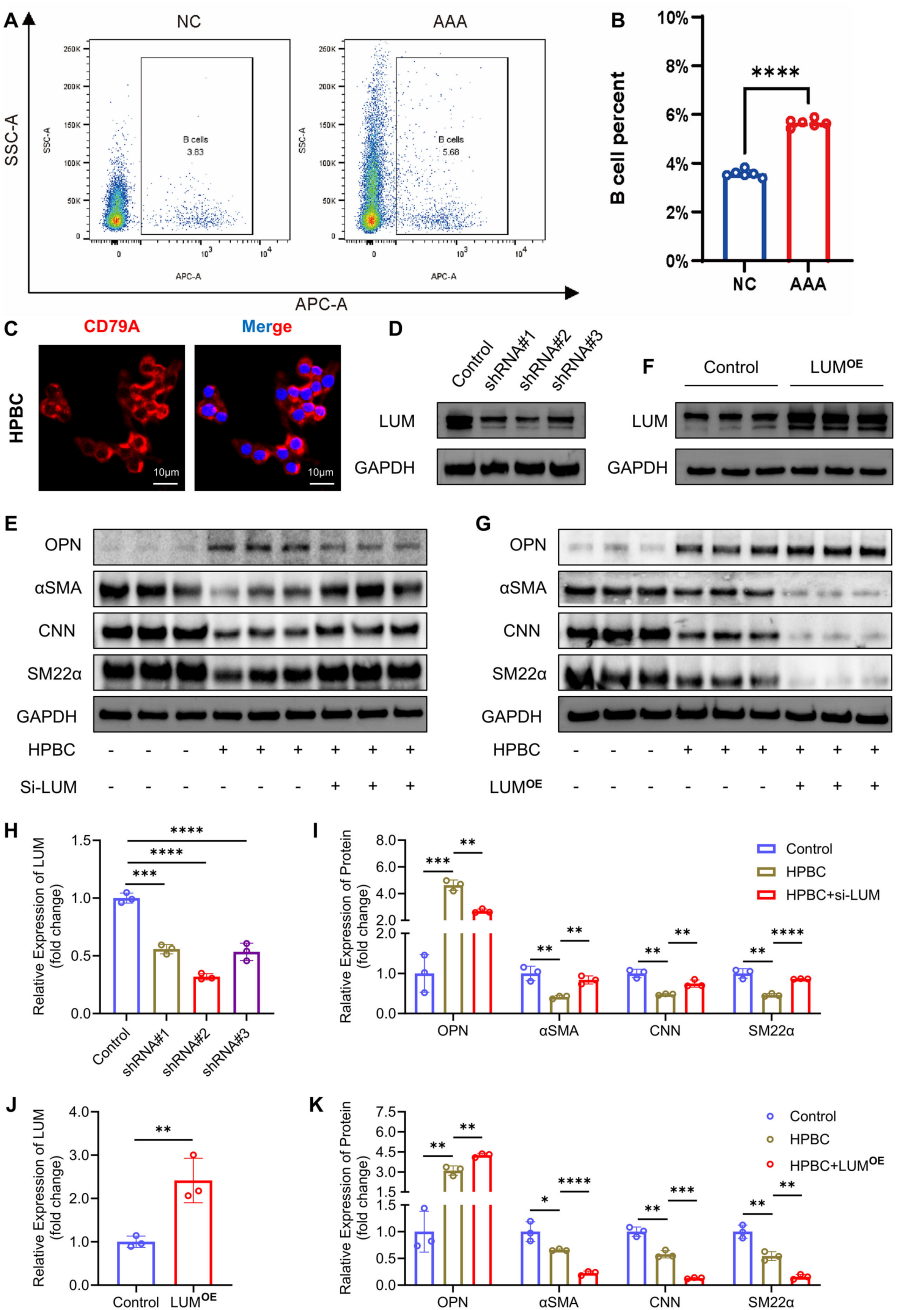
Functional validation of LUM in B cells by lentiviral knockdown and overexpression. **(A)** Representative flow cytometry plots showing the gating strategy for CD79A^+^ B cells isolated from peripheral blood of normal controls (NC) and AAA patients. **(B)** Quantification of the percentage of CD79A^+^ B cells in peripheral blood(n = 6 per group). **(C)** Immunofluorescence staining of primary human peripheral blood B cells (HPBC) confirming B cell identity. Cells were stained for CD79A (red) and counterstained with DAPI (blue). Scale bar = 10 μm. **(D)** Western blot analysis showing LUM protein levels in B cells after lentivirus-mediated shRNA knockdown. **(E)** Western blot analysis of VSMC phenotypic markers (OPN, αSMA, CNN, SM22α) after Transwell co-culture with HPBC in the presence or absence of LUM knockdown. **(F)** Western blot analysis showing LUM protein levels in B cells after lentivirus-mediated overexpression (LUM^OE^). **(G)** Western blot analysis of VSMC phenotypic markers (OPN, αSMA, CNN, SM22α) after Transwell co-culture with HPBC in the presence or absence of LUM overexpression. **(H)** Quantification of LUM protein levels in B cells after lentivirus-mediated shRNA knockdown, corresponding to panel **(D)** (n = 3). **(I)** Quantification of VSMC phenotypic marker expression after co-culture with HPBC ± LUM knockdown, corresponding to panel **(E)** (n = 3). **(J)** Quantification of LUM protein levels in B cells after lentivirus-mediated overexpression, corresponding to panel **(F)** (n = 3). **(K)** Quantification of VSMC phenotypic marker expression after co-culture with HPBC ± LUM overexpression, corresponding to panel **(G)** (n = 3). ^*^p<0.05, ^**^p<0.01, ^***^p<0.001, ^****^p<0.0001.

We first confirmed that the proportion of circulating CD79A^+^ B cells was significantly increased in AAA patients compared with normal controls by flow cytometry (**Figures 5A, B**), and validated the identity of isolated HPBC by immunofluorescence staining for CD79A (**Figure 5C**). Efficient lentiviral shRNA knockdown and overexpression of LUM in B cells were verified by Western blot analysis (**Figures 5D, F, H, J**).

In a Transwell co-culture system, HPBC significantly promoted VSMC phenotypic switching toward a synthetic phenotype, as evidenced by upregulated expression of the synthetic marker OPN and downregulated expression of contractile markers (αSMA, CNN, and SM22α) (**Figures 5E, I**). Importantly, lentivirus-mediated shRNA knockdown of LUM in B cells markedly attenuated HPBC-induced VSMC phenotypic switching (**Figures 5E, I**). In contrast, overexpression of LUM in B cells further aggravated the synthetic phenotypic switch in VSMCs (**Figures 5G, K**).

Collectively, these *in vitro* functional studies demonstrate that LUM expressed in B cells acts as a key mediator driving VSMC phenotypic switching and dysfunction in abdominal aortic aneurysm.

### 3.7 Signaling pathways involved in CD79A and LUM

**Figure 6:**
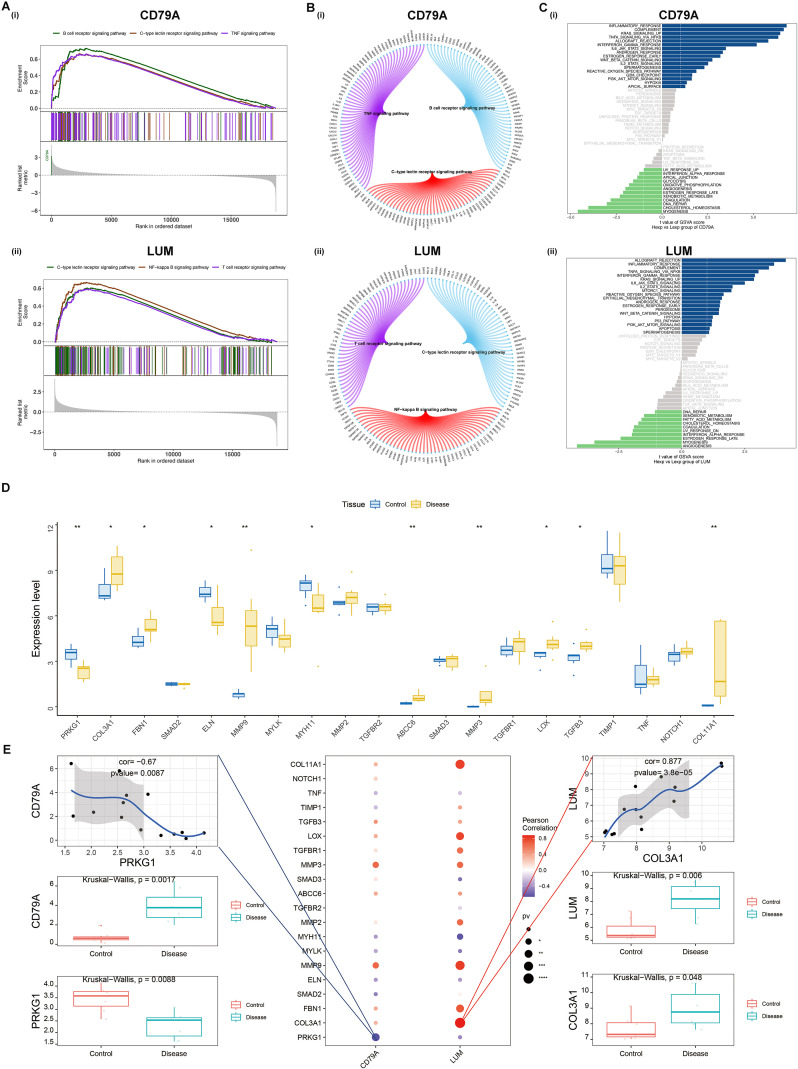
Signaling pathways involved in CD79A and LUM. **(A, B)** GSEA results showed that CD79A was enriched in signaling pathways such as the TNF signaling pathway, the B cell receptor signaling pathway, and the C-type lectin receptor signaling pathway, while LUM was enriched in signaling pathways such as the T cell receptor signaling pathway, the C-type lectin receptor signaling pathway, and the NF-kappa B signaling pathway. **(C)** GSVA analysis showed that CD79A was enriched in signaling pathways such as INFLAMMATORY_RESPONSE, COMPLEMENT, and KRAS_SIGNALING_UP, while LUM was enriched in signaling pathways such as ALLOGRAFT_REJECTION, INFLAMMATORY_RESPONSE, and COMPLEMENT. **(D, E)** Combination of high relevance score and robust expression in transcriptomic data identified CD79A expression demonstrated a strong negative correlation with PRKG1, LUM showed a pronounced positive correlation with COL3A1. *p<0.05, **p<0.01.

Next, we investigated the specific signaling pathways involved in CD79A and LUM to explore the potential molecular mechanisms by which they influence disease progression. Gene Set Enrichment Analysis (GSEA) results showed that CD79A was enriched in signaling pathways such as the TNF signaling pathway, the B cell receptor signaling pathway, and the C-type lectin receptor signaling pathway, while LUM was enriched in signaling pathways such as the T cell receptor signaling pathway, the C-type lectin receptor signaling pathway, and the NF-kappa B signaling pathway (**Figures 6A, B**). Gene Set Variation Analysis (GSVA) showed that CD79A was enriched in signaling pathways such as INFLAMMATORY_RESPONSE, COMPLEMENT, and KRAS_SIGNALING_UP, while LUM was enriched in signaling pathways such as ALLOGRAFT_REJECTION, INFLAMMATORY_RESPONSE, and COMPLEMENT (**Figure 6C**). This suggests that CD79A and LUM may influence disease progression through these pathways.

To further investigate the pathological mechanisms underlying AAA, we retrieved disease-associated genes from the GeneCards database (https://www.genecards.org/). From these, we selected the top 20 genes based on a combination of high relevance score and robust expression in our transcriptomic data for intergroup differential expression analysis. This analysis identified significant expression differences in PRKG1, ELN, MMP9, and COL11A1 between AAA and control groups. Furthermore, correlation analysis between the key genes (CD79A and LUM) and these disease-associated genes revealed statistically significant relationships. Specifically, CD79A expression demonstrated a strong negative correlation with PRKG1 (r = -0.67, p < 0.001), whereas LUM showed a pronounced positive correlation with COL3A1 (r = 0.877, p < 0.001) (**Figures 6D, E**). These robust correlations suggest a potential interaction between CD79A and LUM and established mediators of AAA pathogenesis, particularly implicating LUM in extracellular matrix remodeling processes.

### 3.8 CD79A and LUM expression, co-expression networks, and pathway activity

**Figure 7:**
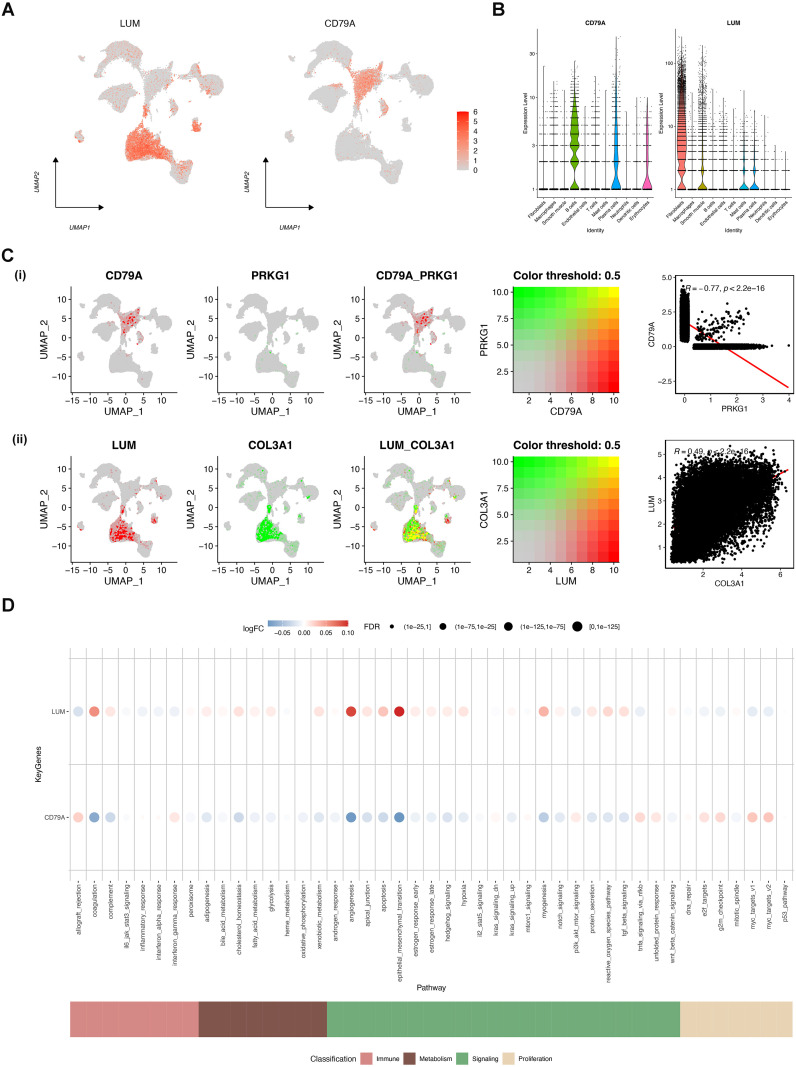
CD79A and LUM expression abundance and co-expression networks, and activity differences with immune/metabolism pathways. **(A)** FeaturePlot visualization of LUM and CD79A expression in UMAP space, revealing enrichment in a discrete B cell-consistent subpopulation. **(B)** VlnPlot analysis showing that CD79A is most highly expressed in B cells, while LUM is elevated in both B cells and fibroblasts. **(C)** The co-expression analysis showing a significant negative correlation between CD79A and PRKG1 (R = −0.77, p < 2.2e−16) and a significant positive correlation between LUM and COL3A1 (R = 0.49, p < 2.2e−16). **(D)** Bubble plots shows that LUM is preferentially active in coagulation, angiogenesis, and epithelial-mesenchymal transition, while CD79A is more active in immune pathways including allograft rejection and complement signaling.

The expression of CD79A and LUM in individual cells was visualized using VlnPlot and FeaturePlot in Seurat (**Figures 7A, B**). We then identified significantly correlated disease genes from the disease correlation analysis and explored the co-expression networks of CD79A and LUM with disease-related genes at the single-cell level through correlation analysis (**Figure 7C**). We then used the AUCell function to quantitatively analyze the single-cell data at the immune and metabolic pathway level, and used bubble plots to visualize the activity differences of CD79A and LUM in immune and metabolic pathways. The results showed that LUM had higher activity in pathways such as coagulation, angiogenesis, and epithelial_mesenchymal_transition, while CD79A had higher activity in pathways such as allograft_rejection, myc_targets_v1, and myc_targets_v2 (**Figure 7D**). These divergent pathway activity profiles further support the distinct functional roles of LUM and CD79A in AAA: LUM appears primarily involved in vascular remodeling and matrix-related processes, while CD79A is more closely linked to immune activation and proliferative signaling.

### 3.9 Computational validation of ligand-protein binding stability

**Figure 8:**
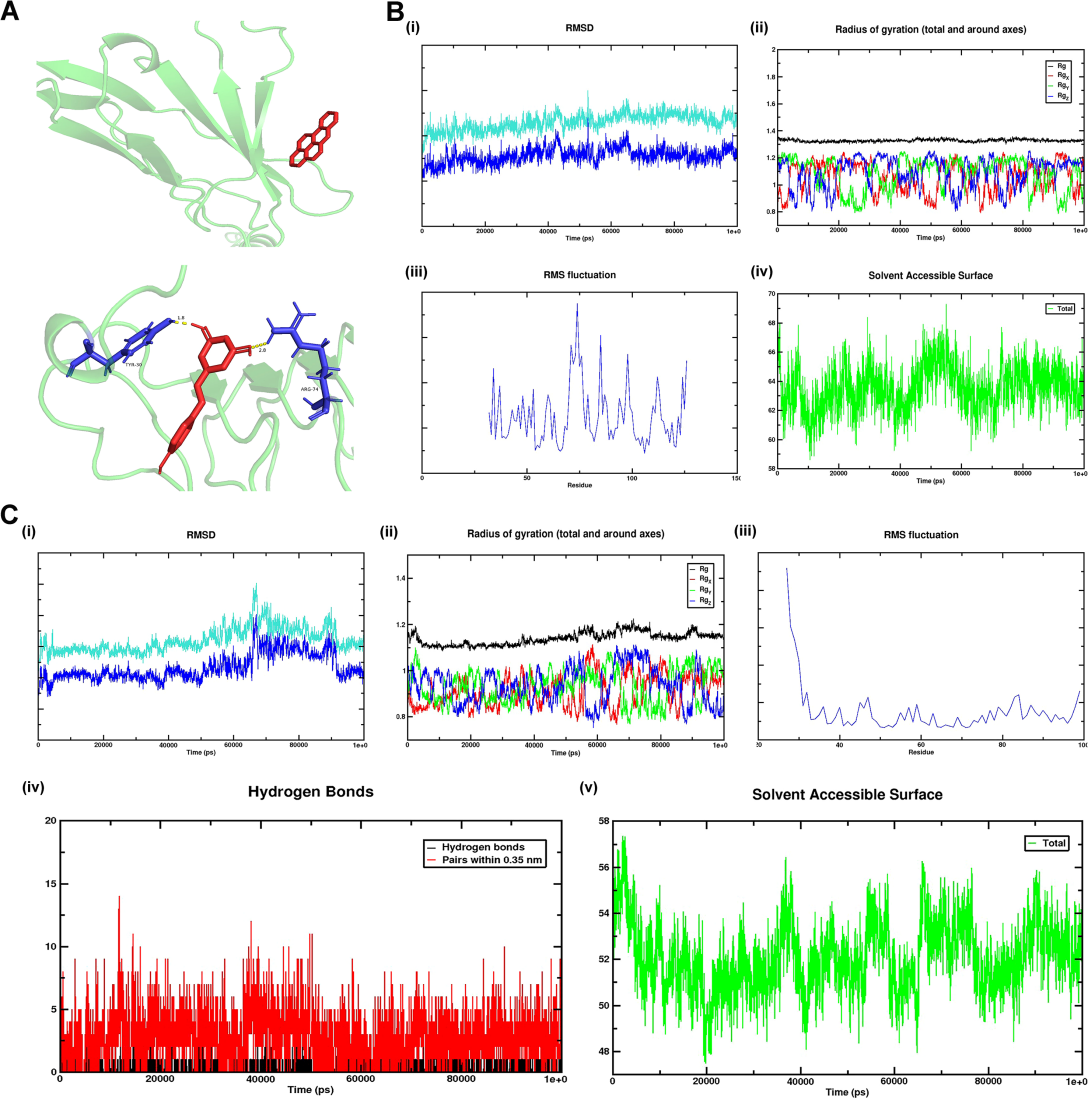
Computational validation of ligand-protein binding stability. **(A)** Molecular docking and molecular dynamics (MD) simulations for the predicted complexes CD79A: Benzo[a]pyrene and LUM: Resveratrol. **(B)** MD simulations confirmed the structural stability of both complexes. **(C)** The consistent number of hydrogen bonds and the stabilization of the buried SASA further indicated a stable binding mode for this complex.

To computationally validate the interactions involving the key genes, we performed molecular docking and molecular dynamics (MD) simulations for the predicted complexes CD79A: Benzo[a]pyrene and LUM: Resveratrol. Docking results demonstrated favorable binding energies of -7.1 kcal/mol and -6.3 kcal/mol for the respective complexes (**Figure 8A**). Subsequent MD simulations confirmed the structural stability of both complexes. For CD79A: Benzo[a]pyrene, the root-mean-square deviation (RMSD) of the complex and protein, the radius of gyration (Rg), and the buried solvent-accessible surface area (SASA) all stabilized over the simulation time, indicating a steady binding interface and complex conformation (**Figure 8B**). Similarly, for LUM: Resveratrol, stable RMSD and Rg values were observed (**Figure 8C**). The consistent number of hydrogen bonds and the stabilization of the buried SASA further indicated a stable binding mode for this complex (**Figure 8C**). Low root-mean-square fluctuation (RMSF) values across most residues in both simulations suggested limited flexible regions and overall protein structural integrity upon ligand binding (**Figures 8B, C**). Collectively, these computational analyses confirm that both small molecules form stable complexes with their respective protein targets, which provides possibility for the future targeted therapy in AAA.

## 4 Discussion

This study integrates single-cell transcriptomics, bulk RNA sequencing, and functional validation experiments to investigate the immune microenvironment of AAA. Our findings indicate that CD79A and LUM are co-expressed in a disease-associated B cell subpopulation. Through lentiviral knockdown and overexpression combined with VSMC co-culture assays, we provide evidence suggesting that LUM in B cells may influence VSMC phenotypic switching. These results offer new cellular and molecular perspectives on B cell-related mechanisms in AAA pathogenesis.

Recent studies have identified that AAA is characterized by immune cells inducing inflammation and immune responses within the adventitia and media of the vascular wall through mechanisms such as antibody production and cytokine secretion (2, 11). Our research corroborates these findings by demonstrating significant accumulation of immune cells, including B cells, plasma cells, macrophages, and neutrophils within AAA tissues, further confirming AAA as a chronic immune inflammatory disease.

Using single-cell RNA sequencing(sc-RNA-seq), we further resolved the spatial and functional heterogeneity of these immune population, First, using MiloR spatial differential abundance analysis, we found that not only is there an increase in the number of B cell subpopulations, but their local spatial distribution also undergoes specific changes, indicating a potential unique intercellular interaction network at the AAA lesion site. CytoTRACE2 and Monocle2 analyses further revealed that B cells in the disease group exhibit lower differentiation potential and a tendency towards a more differentiated mid-to-late state compared to control group B cells. The mid-to-late pseudotemporal state of disease-associated B cells does not appear to reflect terminal plasma cell differentiation: plasma cells formed a distinct cluster (cluster 8) in our single-cell annotation, and the late-phase gene cluster was enriched for ECM-associated genes such as COL6A1 and LUM, with little evidence of classical plasma cell markers such as PRDM1 or IRF4. This transcriptional profile is more consistent with an activated effector or antigen-presenting state, in which B cells likely contribute to disease through cytokine secretion and direct cell-to-cell signaling rather than antibody production. Defining the precise identity of this population will require more granular B cell subtype profiling in future work.

The B cell receptor (BCR) is a hallmark surface complex of B cells that plays a critical role at multiple stages in the B cell lifecycle (26, 27). CD79A is an essential component of BCR, significantly influencing its functionality, signal initiation, and transduction. Our study reveals a significant positive correlation between high expression of CD79A and the infiltration levels of M0 macrophages, neutrophils, and activated T cells. Additionally, GSEA and GSVA indicated that the expression levels of CD79A in TNF, B cell receptor, C-type lectin signaling pathways, and inflammatory responses are significantly increased. This implies that B cells may be activated through persistent or aberrant BCR signaling during the occurrence and progression of AAA, thus acting as amplifiers of local inflammatory responses and recruiting more innate and adaptive immune cells, leading to a vicious cycle (28).

In addition to CD79A, we identified LUM as a critical gene. As a typical extracellular matrix protein, LUM is directly involved in the assembly and stabilization of collagen fibers. Its expression is closely correlated with the collagen gene COL3A1, providing a direct molecular basis for the degradation and remodeling of the extracellular matrix in AAA. Furthermore, we found that LUM is specifically correlated with neutrophil infiltration, suggesting that LUM may regulate the recruitment and function of neutrophils by altering the physicochemical properties of the extracellular matrix or releasing chemokines. The co-expression of CD79A and LUM in B cells raises the possibility that persistently or aberrantly activated B cells with high CD79A expression may participate in the degradation and reconstruction of the extracellular matrix through upregulation of LUM expression, while also influencing other immune cells through cytokine secretion or antibody production.

Moreover, CellChat analysis revealed a significant increase in both the number and strength of intercellular interactions in AAA tissues. Notably, communication between B cells and VSMCs was markedly enhanced, with several ligand-receptor pairs, including TNF–TNFRSF1A, being consistently upregulated across multiple cell pairs. Given that phenotypic switching of VSMCs from a contractile to a synthetic phenotype is a central pathological feature of AAA, we next sought to determine whether LUM expressed in B cells could functionally modulate VSMC behavior. Using lentivirus-mediated shRNA knockdown and overexpression in primary human B cells followed by Transwell co-culture, we demonstrated that B cells promoted VSMC synthetic phenotypic switching, characterized by upregulated OPN and downregulated contractile markers (αSMA, CNN, and SM22α). Importantly, knockdown of LUM in B cells attenuated these phenotypic changes, whereas overexpression of LUM tended to enhance the synthetic phenotype. These findings suggest that LUM in B cells may contribute to VSMC dysfunction in AAA through paracrine signaling. This functional evidence complements our bioinformatic observations and supports the notion that activated B cells act as key regulators in the AAA microenvironment, not only through inflammatory signaling but also by directly influencing vascular smooth muscle cell phenotype.

However, it is important to note that the role of B cells in AAA pathogenesis remains a subject of ongoing debate. Prior studies have reported that adoptive transfer of B2 cells suppresses experimental AAA formation (18) and that anti-CD20-mediated B cell depletion promotes an immunosuppressive aortic microenvironment protective against aneurysm growth (19), suggesting a potentially protective function for certain B cell subsets. These contradictions most likely reflect the functional heterogeneity among B cell subpopulations, wherein effector and regulatory subsets may exert opposing influences depending on disease stage and local tissue context. The B cell subpopulation identified in the present study exhibits a highly activated, differentiated transcriptional state with strong BCR signaling activity, consistent with an effector rather than regulatory phenotype. Due to B cell subsets may carry divergent functional roles in AAA, therapeutic strategies targeting specific molecular features of pathogenic subpopulations, such as CD79A and LUM, may offer greater precision than approaches relying on wholesale B cell depletion.

To validate the potential of CD79A and LUM as biological targets in future clinical applications, we conducted molecular docking and dynamics simulations of their proteins with small molecule ligands. The stable binding conformations, low binding free energies, and robust structures during the 100 ns simulation suggest that small molecule drug design targeting these proteins is structurally feasible. While benzo[a]pyrene and resveratrol serve here as proof-of-concept ligands rather than therapeutic candidates, these results establish a structural basis for future target-directed drug development in AAA.

This study integrates multi-omics approaches, cross-validating single-cell maps with tissue-level bulk sequencing data to enhance the reliability of our conclusions. We employed advanced bioinformatics tools, including spatial differential abundance, developmental potential assessment, and cell communication inference, allowing for a multidimensional deep analysis of cell functions that transcends simple cell type identification. Furthermore, we established a complete logical chain from data mining, key gene screening, pathway enrichment to computational validation, providing clear hypotheses and targets for subsequent experimental research.

In summary, this study highlights the potential importance of B cells and their key molecules, CD79A and LUM, in the immune microenvironment of AAA. By combining multi-omics analyses with *in vitro* functional validation, our findings suggest that LUM expressed in B cells may participate in regulating VSMC phenotypic switching. These observations provide new insights into B cell-mediated mechanisms in AAA and identify LUM as a candidate for further investigation. Future studies using *in vivo* genetic models and larger clinical cohorts will be essential to validate these findings and explore their therapeutic potential.

### 4.1 Limitations

This study has several limitations that should be acknowledged. First, this study is based on available public datasets with limited sample sizes which restricts the generalizability of the findings. Future research should aim to expand the sample size and conduct multi-center validations. Second, single-cell RNA sequencing itself carries technical biases and is not able to directly reflect protein activity and modification states. It should be noted that the conclusions of this study are primarily derived from bioinformatics analyses, which reveal associations rather than establish causality. Third, the molecular docking results reflect computational predictions in the absence of experimental structural data and should be regarded as hypothesis-generating rather than definitive evidence of drug-target interaction. Fourth, although our Transwell co-culture system demonstrated that LUM expression in B cells can influence VSMC phenotypic switching, this *in vitro* model does not fully recapitulate the complex cellular interactions and microenvironment present in AAA tissues *in vivo*. Moreover, the specific paracrine factors or signaling molecules mediating the crosstalk between B cells and VSMCs were not identified in the present study. Future investigations using more advanced co-culture systems, conditioned medium fractionation, or *in vivo* models are warranted to elucidate the precise mechanisms underlying B cell–VSMC communication.

## Data Availability

The datasets presented in this article are not readily available because no. Requests to access the datasets should be directed to song.chen@whu.edu.cn.
