# Supplementary material for: Single-cell sequencing combined with transcriptome analysis unravels LUM+ B cells as key drivers in abdominal aortic aneurysm
Source: Front Immunol. 2026 Jul 1;17:1836487. doi: 10.3389/fimmu.2026.1836487 (PMC13368562; doi:10.3389/fimmu.2026.1836487)
Supplement: Supplementary file 1 [file DataSheet1.docx]

**Supplemental Materials**


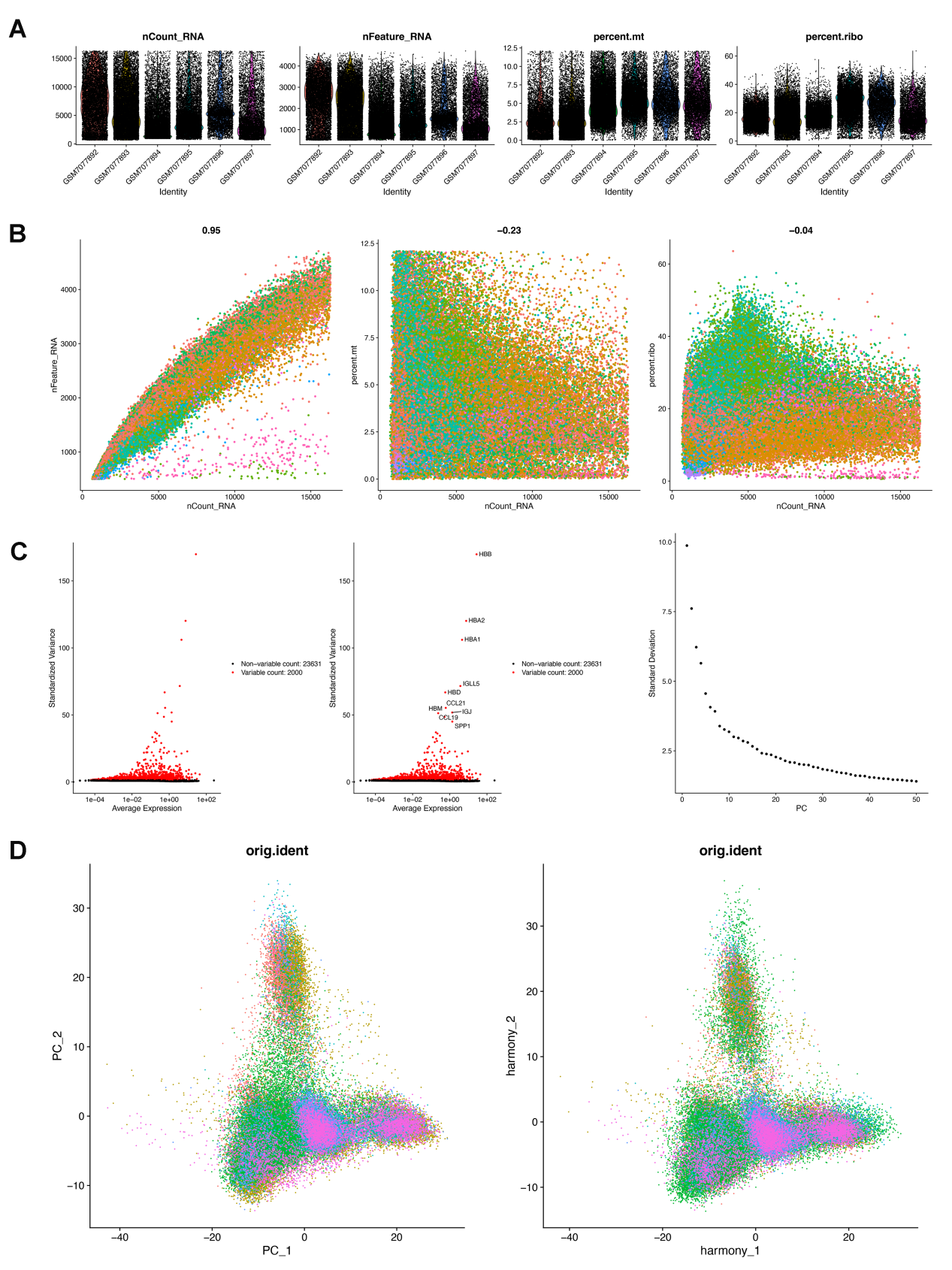


**Supplementary Fig. 1. Single-cell data quality control, dimensionality reduction**. (A) Violin plots after quality control; (B) Scatter plots after quality control; (C) Principal component analysis and batch effect correction; (D) UMAP plot.

**Supplementary Table S1. List of primers for qRT-PCR.**

| **Gene name** | **Symbol** | **Forward primer** | **Reverse primer** | **Annealing Temperature (°C)** |
| --- | --- | --- | --- | --- |
| Glyceraldehyde-3-phosphate dehydrogenase | *GAPDH* | GAAGGTGAAGGTCGGAGTC | GAAGATGGTGATGGGATTTC | 56 |
| [Lumican](https://www.ncbi.nlm.nih.gov/datasets/gene/4060) | *LUM* | TAACTGCCCTGAAAGCTACCC | GGAGGCACCATTGGTACACTT | 56 |

**Supplementary Table S2. List of primary antibodies.**

| **Antigens** | **Species antibodies raised in** | **Dilution (WB)** | **Dilution (IHC)** | **Supplier** |
| --- | --- | --- | --- | --- |
| GAPDH, human | Mouse, monoclonal | 1:2,000 | - | Santa Cruz Biotechnology Inc., USA, Cat. #sc-365062 |
| LUM, human | Rabbit, Polyclonal | 1:500 | 1:100 | Proteintech, China, Cat. #10677-1-AP |
| CD79a, human | Rabbit, Polyclonal | - | - | Proteintech, China, Cat. #122349-1-AP |
| CD79a, human(FC) | Rabbit, Polyclonal | - | - | Proteintech, China, Cat. #APC-FcA98072 |
| αSMA, human | Rabbit, Polyclonal | 1:1000 | - | Proteintech, China, Cat. #14395-1-AP |
| SM22, human | Rabbit, Polyclonal | 1:1000 | - | Proteintech, China, Cat. #10493-1-AP |
| OPN, human | Rabbit, Polyclonal | 1:1000 | - | Proteintech, China, Cat. #22952-1-AP |
| Calponin,human | Rabbit, Polyclona | 1:1,000 | - | Abclonal,China, Cat. #A16638 |

**Supplementary Table S3. List of secondary antibodies and counterstaining of nuclei.**

| **Secondary detection system used** | **Host** | **Method** | **Dilution** | **Supplier** |
| --- | --- | --- | --- | --- |
| Anti-Mouse-IgG (H+L)-HRP | Goat | WB | 1:10,000 | Sungene Biotech, China, Cat. #LK2003 |
| Anti-Rabbit-IgG (H+L)-HRP | Goat | WB | 1:10,000 | Sungene Biotech, China, Cat. #LK2001 |
| Hoechst 33342 nucleic acid staining (DAPI) | - | IF | 1:750 | Molecular Probes/Invitrogen, USA, Cat. #A11007 |
