# Supplementary material for: Single-cell sequencing combined with transcriptome analysis unravels LUM+ B cells as key drivers in abdominal aortic aneurysm
Source: Front Immunol. 2026 Jul 1;17:1836487. doi: 10.3389/fimmu.2026.1836487 (PMC13368562; doi:10.3389/fimmu.2026.1836487)
Supplement: Supplementary file 2 [file DataSheet2.docx]

**Original image files for the blots**

**Figure 4. F**

*
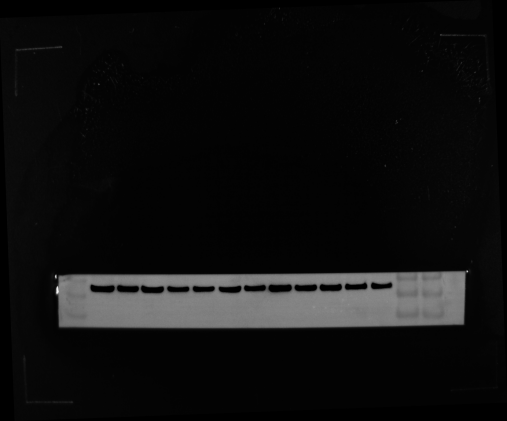

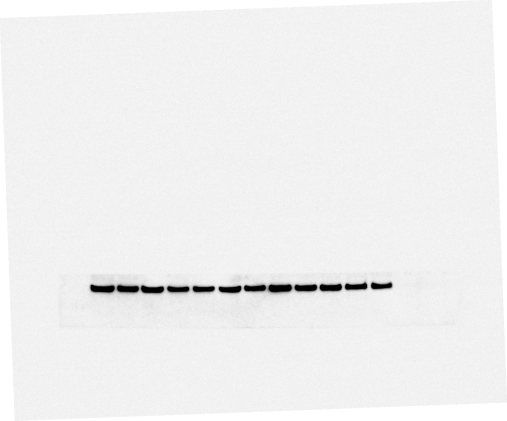
*

**GAPDH**


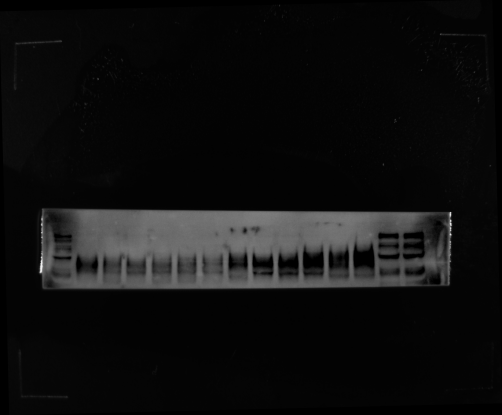

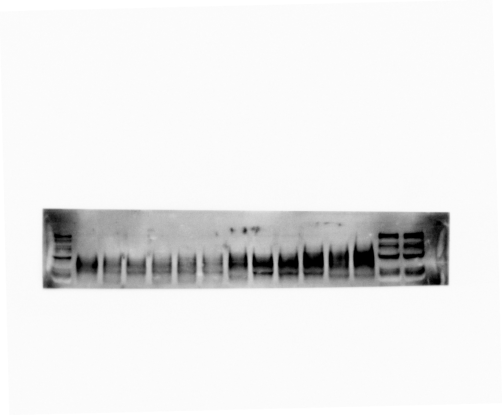


**LUM**

**Figure 5. D**

**
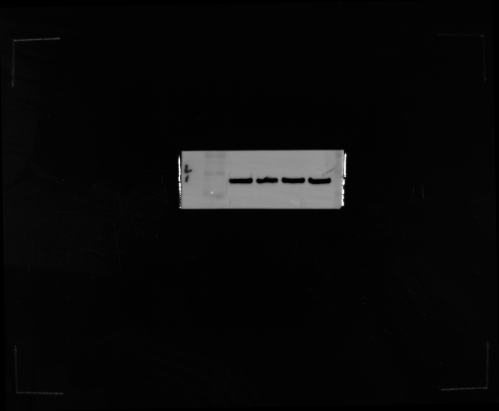

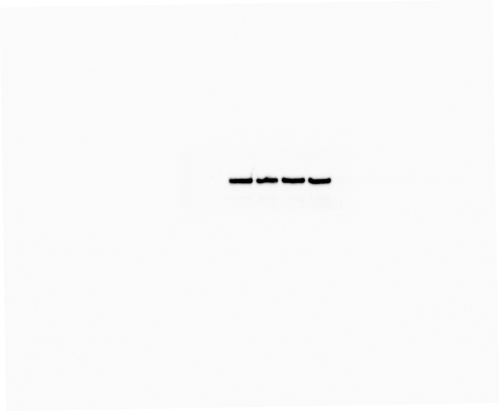
**

**GAPDH**

**
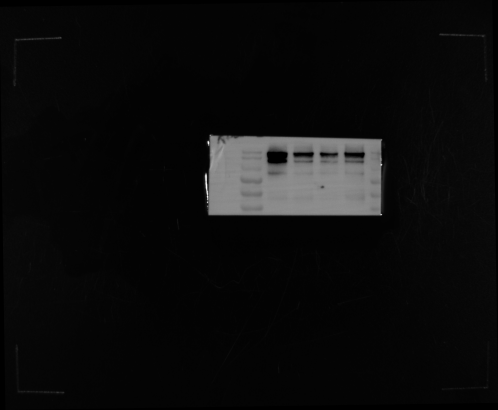

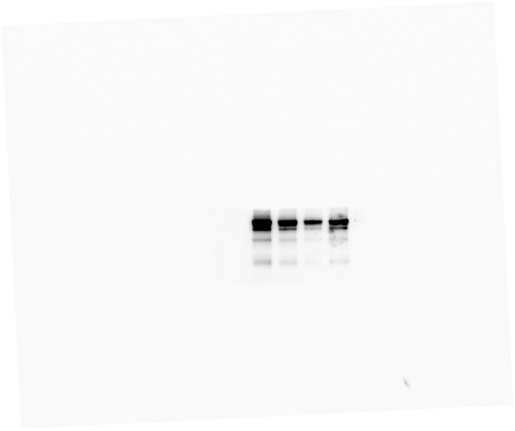
**

**LUM**

**Figure 5. F**

**
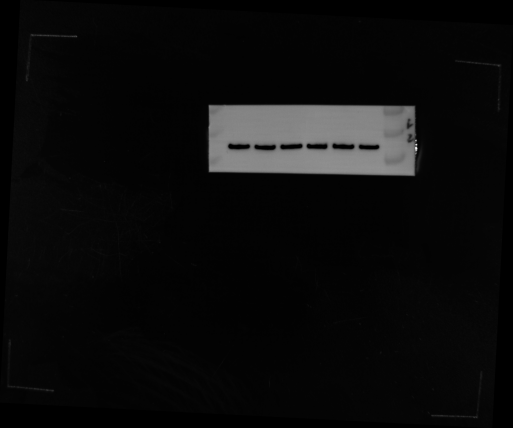

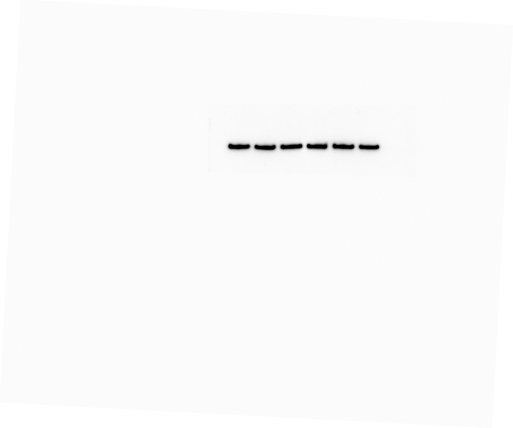
**

**GAPDH**

**
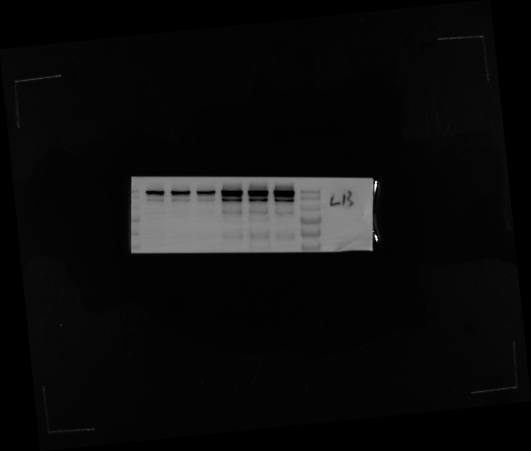

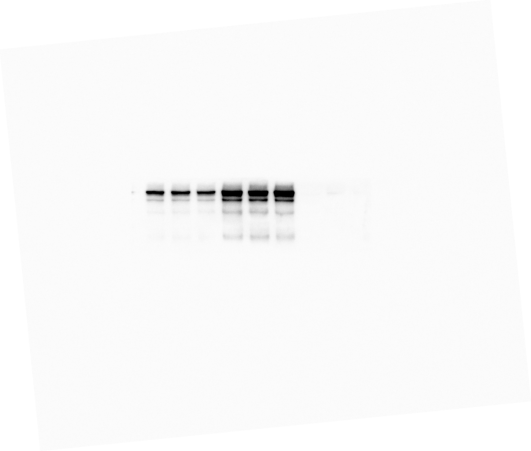
**

**LUM**

**Figure 5. E**

**
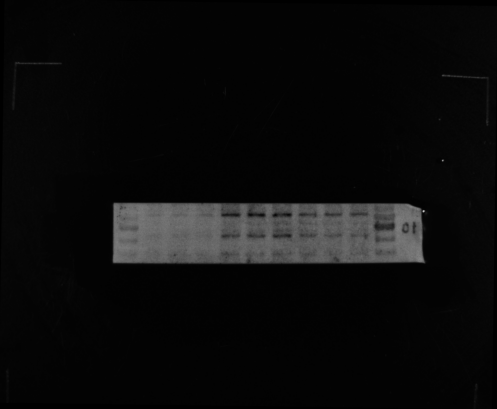

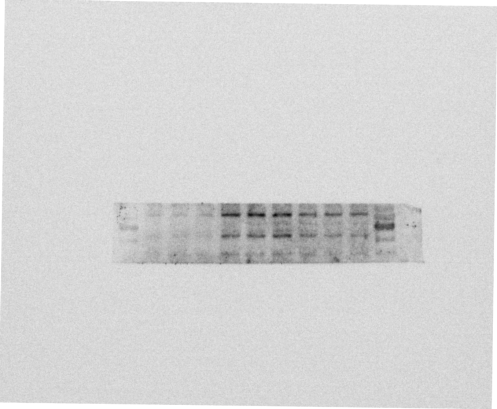
**

**OPN**

**
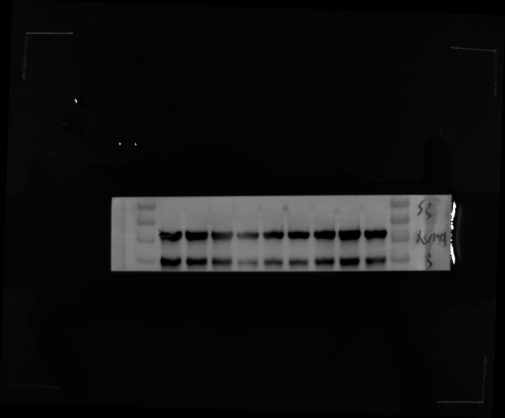

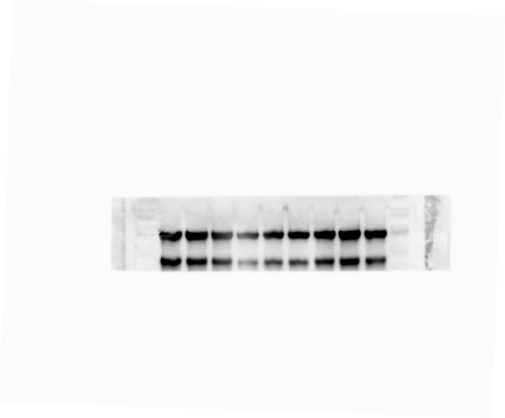
**

**αASMA**

**
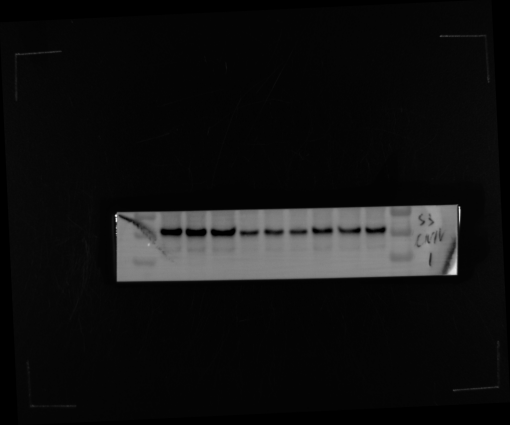

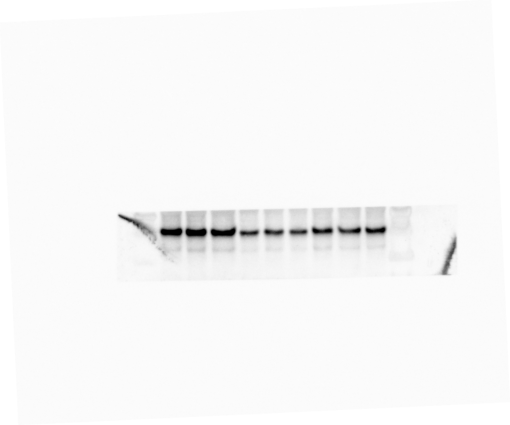
**

**CNN**

**
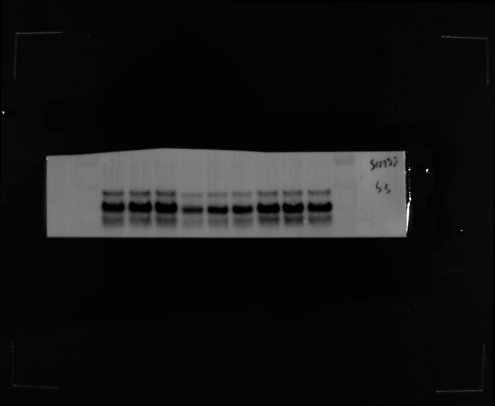

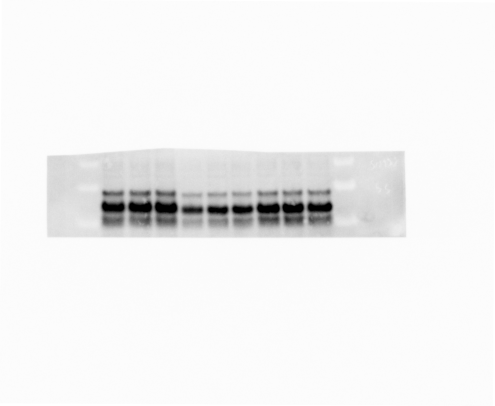
**

**SM22α**

**
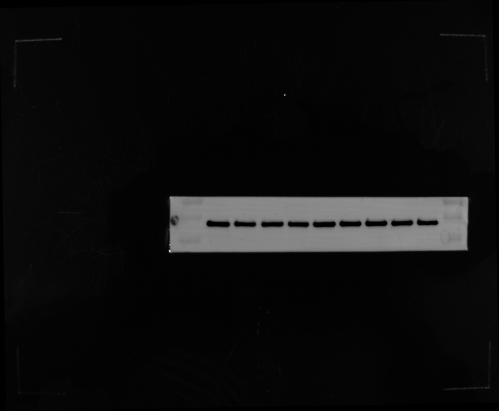

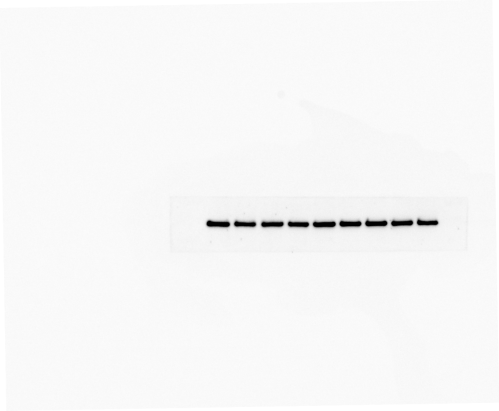
**

**GAPDH**

**Figure 5. G**

**
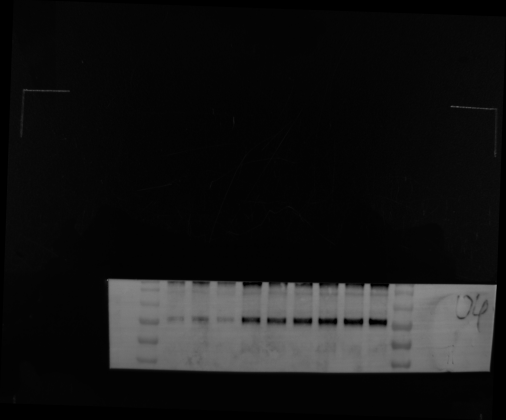

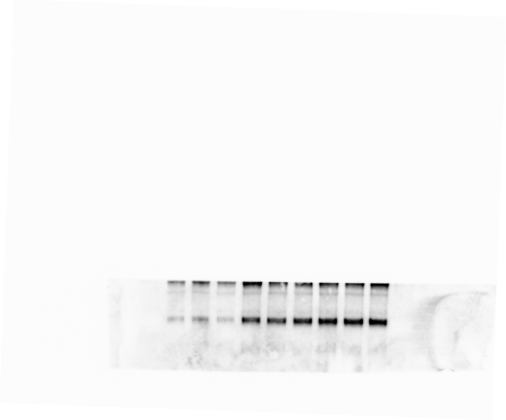
**

**OPN**

**
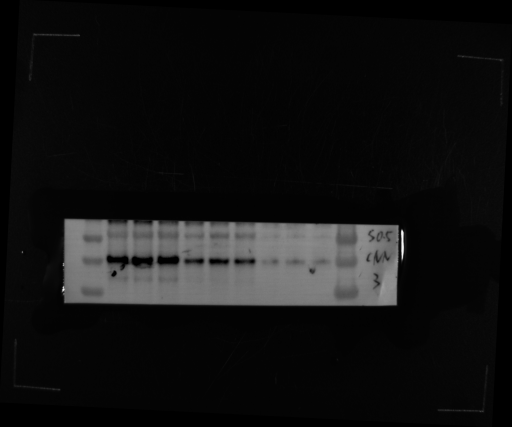

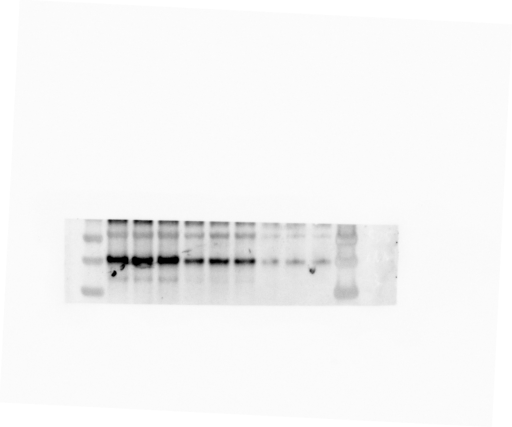
**

**CNN**

**
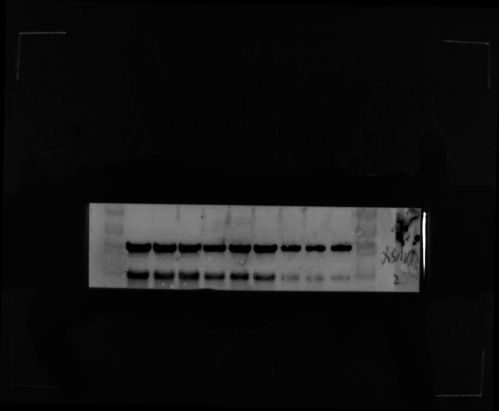

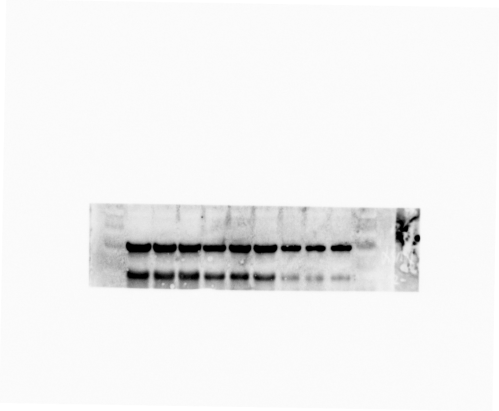
**

**αSMA**

**
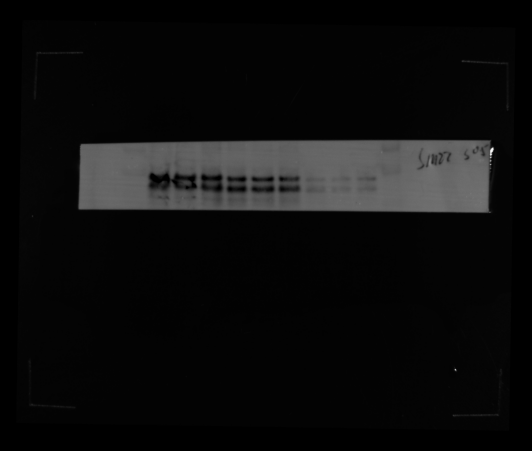

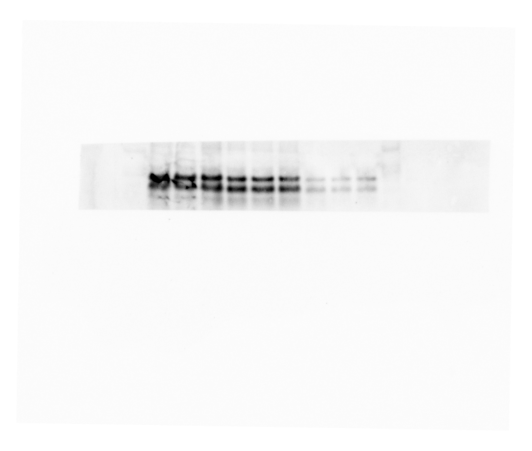
**

**SM22α**

**
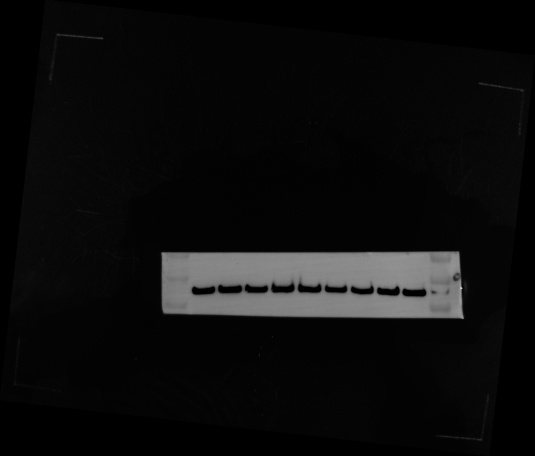

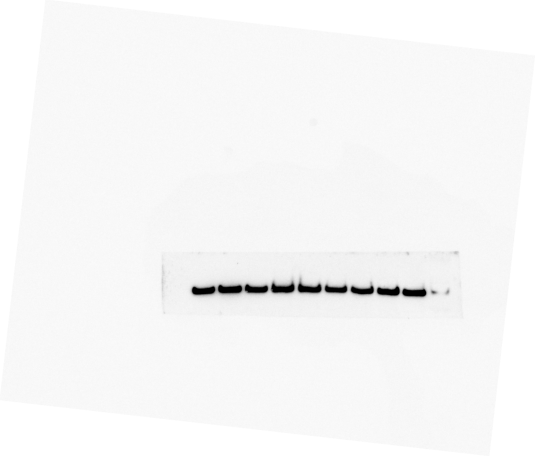
**

**GAPDH**
